# Syncope or seizure: that is the question—case report of a young patient with convulsive cardioinhibitory syncope treated with cardioneuroablation

**DOI:** 10.1093/ehjcr/ytae256

**Published:** 2024-05-16

**Authors:** Andrea Papa, Urs Fisch, Stefano Bassetti, Patrick Badertscher, Philipp Krisai

**Affiliations:** Department of Cardiology and Cardiovascular Research Institute Basel, University Hospital Basel, Basel, Switzerland; Department of Neurology, University Hospital Basel, Basel, Switzerland; Division of Internal Medicine, University Hospital Basel and University of Basel, Basel, Switzerland; Department of Cardiology and Cardiovascular Research Institute Basel, University Hospital Basel, Basel, Switzerland; Department of Cardiology and Cardiovascular Research Institute Basel, University Hospital Basel, Basel, Switzerland

**Keywords:** Case report, Reflex cardioinhibitory syncope, Seizure, Ictal asystole, Cardioneuroablation

## Abstract

**Background:**

Differentiation of syncope from seizure is challenging and has therapeutic implications. Cardioinhibitory reflex syncope typically affects young patients where permanent pacing should be avoided whenever possible. Cardioneuroablation may obviate the need for a pacemaker in well-selected patients.

**Case summary:**

A previously healthy 24-year-old woman was referred to the emergency department after recurrent episodes of transient loss of consciousness (TLOC). The electrocardiogram (ECG) and the echocardiogram were normal. An electroencephalogram (EEG) showed intermittent, generalized pathological activity. During EEG under photostimulation, the patient developed a short-term TLOC followed by brachial myocloni, while the concurrent ECG registered a progressive bradycardia, which turned into a complete atrioventricular block and sinus arrest with asystole for 14 s. Immediately after, the patient regained consciousness without sequelae. The episode was interpreted as cardioinhibitory convulsive syncope. However, due to the pathological EEG findings, an underlying epilepsy with ictal asystole could not be fully excluded. Therefore, an antiseizure therapy was also started. After discussing the consequences of pacemaker implantation, the patient agreed to undergo a cardioneuroablation and after 72 h without complications, she was discharged home. At 10 months, the patient autonomously discontinued the antiepileptics. The follow-up EEG displayed unspecific activities without clinical correlations. An implantable loop recorder didn’t show any relevant bradyarrhythmia. At 1-year follow-up, the patient remained asymptomatic and without syncopal episodes.

**Discussion:**

Reflex syncope must be considered in the differential diagnosis of seizures. The cardioneuroablation obviated the need for a pacemaker and allowed for the withdrawal of anticonvulsants, originally started on the premise of seizure.

**Learning points**

- Convulsive reflex syncope has to be considered in the differential diagnosis of non-epileptic paroxysmal seizures.
- Electrocardiogram and electroencephalogram (EEG) during seizure activity need careful interpretation.
- Cerebral hypoperfusion due to cardioinhibition might cause non-specific EEG activity misleading to a diagnosis of a primary epileptic disease.
- Cardioneuroablation is a novel therapeutic approach with promising results in selected patients with recurrent reflex syncope.

## Introduction

Reflex syncope is the most common cause of syncope and a frequent reason for emergency visits. Given its benign nature, education and reassurance are the cornerstones of management. Convulsive movements may appear during the transient loss of consciousness (TLOC), leading in some cases to a misdiagnosis of seizure, which has therapeutic implications. In patients with severe or recurrent forms of syncope, additional treatment may be necessary. Pacemaker implantation should be considered in patients older than 40 years with recurrent cardioinhibitory reflex syncope. However, in well-selected younger patients, cardioneuroablation (CNA) is emerging as an alternative strategy. We present a case of a patient with cardioinhibitory reflex syncope treated with CNA.

## Summary figure

**Figure:**
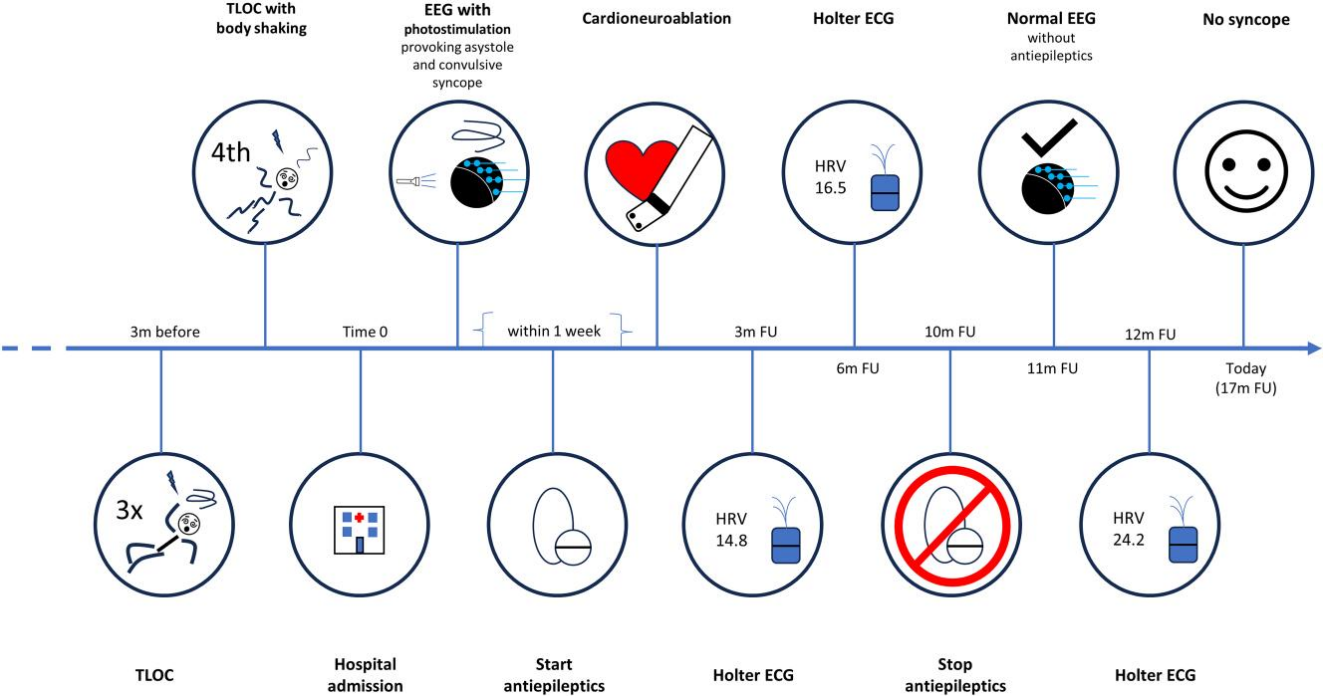
EEG, electroencephalogram; ECG, electrocardiogram; HRV, heart rate variability; FU, follow-up; m, months; TLOC, transient loss of consciousness.

## Case summary

A previously healthy 24-year-old woman was admitted to our hospital after four episodes of TLOC of unknown origin within 3 months. The episodes were mostly situational, while showering or hair-combing with full alertness and awareness afterwards, suggesting a vagal aetiology. No other syncopal episodes were reported in the history. The patient was not taking any prescribed medication at the time of admission, and the family history was negative for sudden cardiac arrest.

The physical examination revealed normal heart sounds, and the blood pressure and the heart rate were within the normal range. The neurological assessment did not reveal any sensory or motor deficits and an orthostatic challenge was conducted, which showed no significant drop in blood pressure. The electrocardiogram (ECG) and the laboratory findings on admission were unremarkable. The echocardiography showed a structurally normal heart.

**Figure 1:**
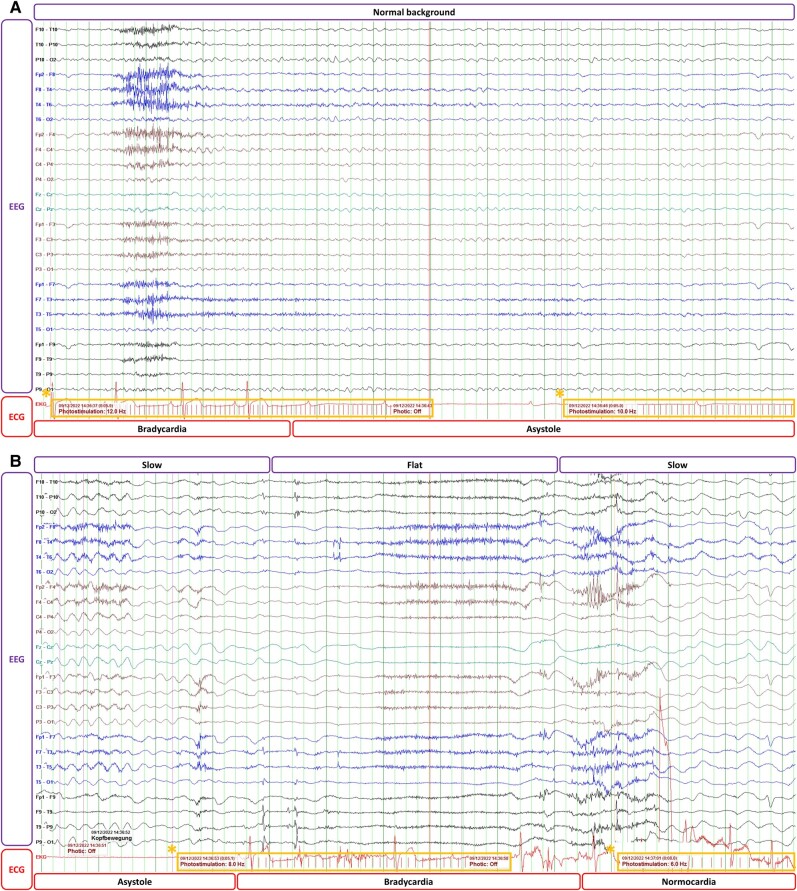
EEG and ECG during photostimulation. (*A*) The ECG shows the beginning of asystole while the EEG is still normal. (*B*) The EEG shows a slow-flat-slow pattern after the 14 s of asystole. The asterisks (*) mark the train of light stimuli (photostimulation).

**Figure 2:**
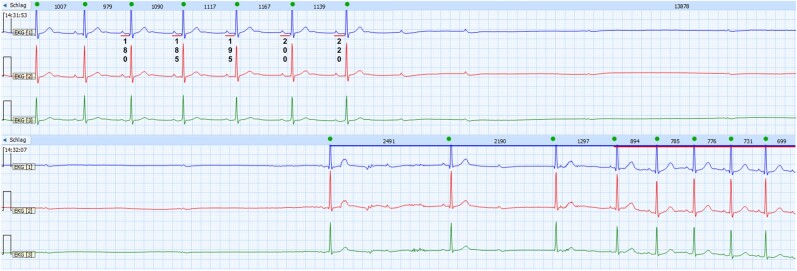
Analysis of the ECG during photostimulation. The ECG shows progressive RR interval prolongation and PR prolongation before complete atrioventricular block with sinus arrest.

Due to shaking arms and tongue biting during the last episode, the patient was also evaluated for possible epilepsy. The brain magnetic resonance imaging did not show any relevant structural abnormalities. The first electroencephalogram (EEG) at rest showed pathological repetitive short epochs of high amplitude generalized slowing. To address this, an EEG under photostimulation was performed. During photostimulation, the simultaneously registered ECG showed a progressive bradycardia, which turned into a complete atrioventricular block and sinus arrest with asystole for 14 s. The patient developed a TLOC followed by three bilateral brachial myoclonia. After the complete atrioventricular block, the EEG showed during the TLOC a generalized slow-fast-slow pattern (*Figures 1* and *2*). Thereafter, the patient immediately regained consciousness without sequelae.

The differential diagnoses included epilepsy with ictal asystole or a convulsive reflex syncope. A psychogenic form of TLOC was excluded based on the ECG and EEG findings. We interpreted the episode most likely as a cardioinhibitory convulsive syncope, due to the sequence of asystole and EEG findings typical of cerebral anoxia ending with TLOC. However, due to the repetitive pathological findings in the EEG at rest, an underlying epilepsy could not be fully excluded at the time. Therefore, we started an additional antiseizure therapy with levetiracetam, followed by lamotrigine.

Considering the risk of repetitive asystole, pacemaker implantation was initially discussed. However, this strategy was considered inappropriate for a young patient due to the potential long-term risks such as repeated device replacements, risk for lead failures, and infections. As an alternative, a CNA was discussed with the patient, who agreed to undergo the procedure. This was performed over a biatrial, transseptal approach.

**Figure 3:**
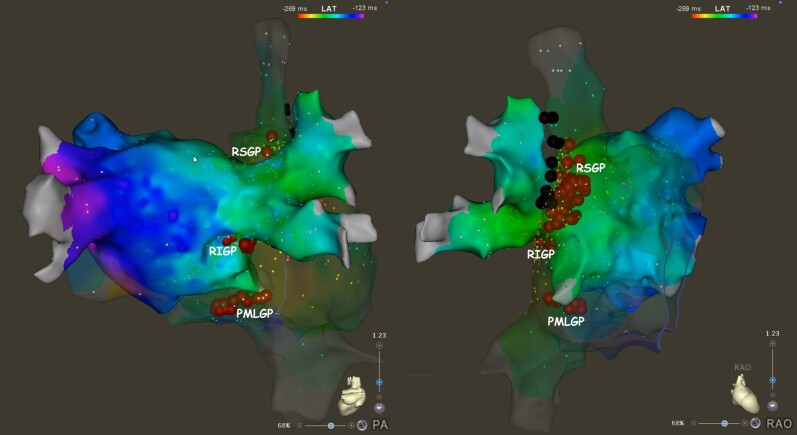
3D electroanatomical mapping of the atria with ablation points and localization of ganglionated plexi. The red tags denote the ablation points. The black tags denote the course of the right phrenic nerve. PA, postero-anterior view; PMLGP, posteromedial left ganglionated plexus; RAO, right anterior oblique view; RIGP, right inferior ganglionated plexus; RSGP, right superior ganglionated plexus.

In the left atrium, we targeted the right superior ganglionated plexus (GP) anterior to the right superior pulmonary vein and the right inferior GP adjacent to the interatrial groove. These GPs were re-ablated from the right atrium with additional ablation at the right atrial/superior vena cava junction and of the posteromedial left GP posteriorly to the coronary sinus ostium under close monitoring of the phrenic nerve (*Figure 3* and Supplementary material online, *Videos S1*–*S3*). A loop recorder was implanted after ablation. After a 72 h observation period without complications, the patient was discharged with a prescription of oral anticoagulation for 2 months and proton pump inhibitor for 6 weeks in addition to the antiepileptic therapy.

**Figure 4:**
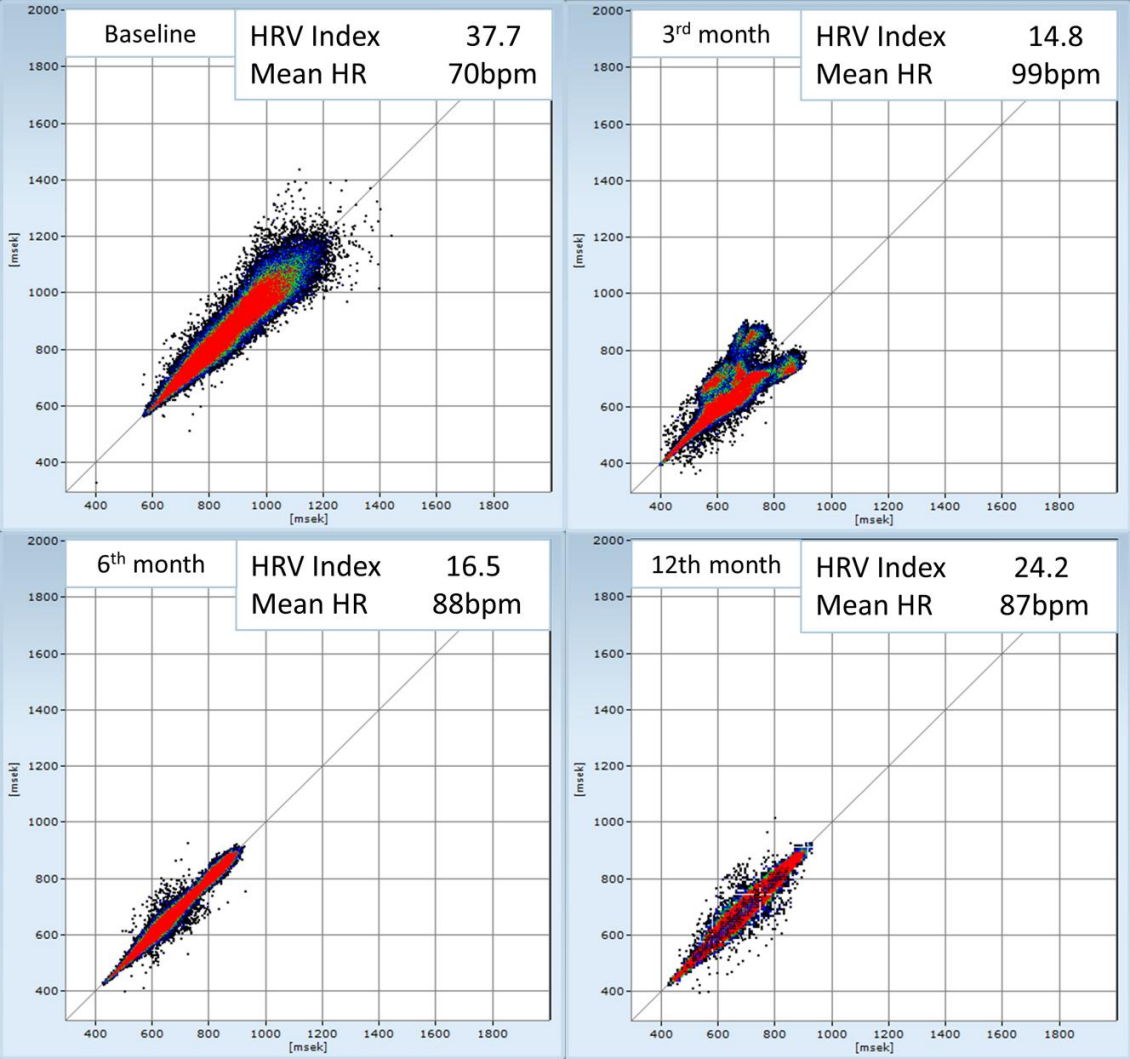
Heart rate variability index and mean heart rate at baseline, 3rd, 6th, and 12th month after ablation. HR, heart rate; HRV, heart rate variability.

A 24 h Holter ECG at 3 months showed a reduced heart rate (HR) variability—index (from 37.7 to 14.8) with a mean HR increase from 70 to 99 b.p.m. suggesting that the ablation was effective in reducing cardiac vagal tone. After 6 and 12 months, the Holter ECGs showed a trend to higher HR variability with a lower mean HR, suggesting a possible reinnervation of some GPs (*Figure 4*). The EEG at 2.5 months after CNA showed very rarely short epochs of focal slowing in the right parieto-occipital leads. The patient autonomously opted to discontinue the antiseizure medication without medical guidance at 10 months. The EEG follow-up at 11 months continued to display short epochs of paroxysmal generalized slowing without clinical correlations and absence of unambiguous epileptiform discharges. After 1 year of follow-up, the patient remained asymptomatic, without syncopal episodes.

## Discussion

Differentiating between convulsive reflex syncope and ictal asystole is fundamental for subsequent treatment decisions. Patient history can provide useful details, like the presence and nature of triggers and prodromes or the duration of unconsciousness and of the confusion after the episode. Myoclonic jerks during syncope can lead to a misdiagnosis of seizure. These movements are usually few, irregular, and asymmetrical in syncope. According to current European guidelines,^1^ due to its low diagnostic yield, EEG should not be routinely used in the assessment of TLOC when syncope is the most likely cause, as a normal resting EEG cannot rule out epilepsy and this may trigger other examinations and generate higher healthcare costs. However, when clinical data are equivocal, an EEG can be useful to distinguish among syncope, pseudosyncope, and epilepsy. During seizures, typical EEG-discharges are recorded, whereas during syncope, the EEG generally shows diffuse slow activity and flattening up to an isoelectric EEG,^2^ as was in our case (*Figure 1B*). Due to the limited number of reported cases of syncope induced by light stimulation, the role of photostimulation during EEG in the context of reflex syncope remains unclear. Tilt testing is also useful to demonstrate susceptibility of the patient to reflex syncope and may be helpful in separating syncope with abnormal movements from epilepsy.^3^

Usually, medical treatment for reflex syncope is not required and reassuring the patient is sufficient. However, when syncopal events recur and impair quality of life, therapeutic options like counter pressure manoeuvers, salt and fluid intake, midodrine or fludrocortisone may be considered.^1^ In case of cardioinhibitory reflex syncope, cardiac pacing should only be considered in highly selected patients i.e. those >40 years of age with recurrent episodes and high risk of injury. In younger patient populations (≤40 year), there is currently insufficient evidence for the use of pacing.^4^

Since 2005, CNA has emerged as a new treatment for vasovagal cardioinhibitory syncope.^5^ By endocardial radiofrequency ablation in the area of GPs, CNA reduces vagal tone and reflectory cardioinhibition. Different ablation strategies, including anatomically guided, electrogram guided, bi-, and only right-atrial have been described, with syncope free survival rates of 6.9–10.6% in observational studies.^6^ Recently, the first randomized control trial compared CNA and optimal non-pharmacological treatment in young patients with cardioinhibitory reflex syncope. After 2 years of follow-up, syncope recurrence in the CNA group was significantly lower than the control group (8% vs. 54%; *P* = 0.0004).^7^ However, multicentre, long-term studies with sham controls are still needed to investigate the role of vagal reinnervation.

## Supplementary Material

ytae256_Supplementary_Data

## Data Availability

The data underlying this article are available in the article and in its online Supplementary material.

## Lead author biography

Andrea Papa is a cardiology resident at the Heart Center of the University Hospital Basel with a broad interest in different subspecialties.

## Notes

**Consent:** The patient undersigned a consent form for the anonymous publication of images and/or information about the case according to the COPE guidelines. Open and continuous communication with the patient allowed a trusting relationship with the physician and the patient never felt exposed to unnecessary risks.

**Funding:** This research received no external funding.
